# Function, essentiality, and expression of cytochrome P450 enzymes and their cognate redox partners in *Mycobacterium tuberculosis:* are they drug targets?

**DOI:** 10.1007/s00253-019-09697-z

**Published:** 2019-02-27

**Authors:** Sandra Ortega Ugalde, Maikel Boot, Jan N. M. Commandeur, Paul Jennings, Wilbert Bitter, J. Chris Vos

**Affiliations:** 1 Division of Molecular Toxicology, Amsterdam Institute for Molecules Medicines and Systems (AIMMS), Faculty of Sciences, Vrije Universiteit, De Boelelaan 1108, 1081 HZ Amsterdam, The Netherlands; 2 Department of Microbial Pathogenesis, Yale University School of Medicine, New Haven, CT USA; 3 Section of Molecular Microbiology, AIMMS, Faculty of Sciences, Vrije Universiteit, Amsterdam, The Netherlands

**Keywords:** Cytochrome P450, *Mycobacterium tuberculosis*, Redox partners, Essentiality, Stress response, Antibiotic exposure response

## Abstract

This review covers the current knowledge of the cytochrome P450 enzymes (CYPs) of the human pathogen *Mycobacterium tuberculosis* (Mtb) and their endogenous redox partners, focusing on their biological function, expression, regulation, involvement in antibiotic resistance, and suitability for exploitation as antitubercular targets. The Mtb genome encodes twenty  CYPs and nine associated redox partners required for CYP catalytic activity. Transposon insertion mutagenesis studies have established the (conditional) essentiality of several of these enzymes for in vitro growth and host infection. Biochemical characterization of a handful of Mtb CYPs has revealed that they have specific physiological functions in bacterial virulence and persistence in the host. Analysis of the transcriptional response of Mtb CYPs and redox partners to external insults and to first-line antibiotics used to treat tuberculosis showed a diverse expression landscape, suggesting for some enzymes a potential role in drug resistance. Combining the knowledge about the physiological roles and expression profiles indicates that, at least five Mtb CYPs, CYP121A1, CYP125A1, CYP139A1, CYP142A1, and CYP143A1, as well as two ferredoxins, FdxA and FdxC, can be considered promising novel therapeutic targets.

## Introduction

### Tuberculosis

Tuberculosis (TB) is caused by the human pathogen *Mycobacterium tuberculosis* (Mtb). In 2017, 10 million new TB cases were reported worldwide and 1.3 million people died from TB infection, making it the deadliest infectious disease known to humankind (World Health Organization 2018). The emergence of Mtb strains resistant to the first-line anti-TB agents*,* i.e., ethambutol, isoniazid, pyrazinamide, and rifampicin, as well as the fatal synergy with HIV, has prompted the development and FDA approval of new drugs for TB, such as bedaquiline and delamanid. In this context, the determination of the complete genome sequence of Mtb and development of genetic tools have been instrumental in uncovering the biology of this unconventional pathogen and the identification of novel drug targets (Cole et al. 1998).

The mycobacterial genome encodes a large variety of enzymes involved in lipid biosynthesis and metabolism, reflecting its elaborate capability to use lipid metabolism for both energy homeostasis and synthesis of many different components of the complex cell envelope (Gazaei 2018). This unusual cell envelope is critical for Mtb’s persistence, providing not only mechanical strength and protection to hostile environments but also a barrier to the entry of harmful compounds (Nikaido and Jarlier 1991; Chiaradia et al. 2017). Fortunately, because the mycobacterial cell envelope contains many unique structures, the enzymes involved in its biogenesis provide us with many Mtb-specific drug targets and thus the possibility to develop compounds that are highly specific to mycobacteria. Recent examples include the benzothiazinones that block arabinogalactan biosynthesis and delamanid that interferes with mycolic acid biosynthesis (Riccardi and Pasca 2014).

Genome mapping revealed that Mtb encodes a high number of cytochrome P450 enzymes (CYPs or P450s). CYPs are haem-containing monooxygenase enzymes involved in lipid and steroid metabolism in eukaryotes. In fungi, several CYPs are already validated drug targets. An example is CYP51, the fungal sterol-14α-demethylase involved in the biosynthesis of membrane sterols. CYP51 can be inhibited by several azole-compounds (Warrilow et al. 2013). The use of anti-fungal azoles to inhibit CYP51 has also been proposed as treatment for parasitic infections, such as Chagas disease and the African sleeping sickness (Lepesheva et al. 2008; Chen et al. 2010). A homolog of CYP51 is also encoded in Mtb genome, *cyp51b1*, highlighting its potential use as a drug target. Interestingly, CYP-inhibiting azole drugs have potent anti-mycobacterial activity and econazole reduced bacterial burden by 90% in the lungs and spleen of mice infected with Mtb (Guardiola-Diaz et al. 2001; Ahmad et al. 2006a, b; McLean et al. 2006a). However, it remains unclear whether the growth inhibition was due to inhibition of one or more Mtb CYPs by econazole (Ahmad et al. 2006b).

In light of the potential role of CYPs in physiology and pathology, as well as their proven efficiency as therapeutic targets in mycobacteria, in-depth understanding of the physiological function and biochemical characterization of these enzymes is a priority. In this review, we update the current knowledge on the essentiality and physiological function of Mtb CYPs and their cognate redox partners, which has been reviewed before, adding new sections on CYP139A1 and CYP143A1 (McLean et al. 2006a, 2007, 2010; McLean and Munro 2008; Ouellet et al. 2010a; Ortiz de Montellano 2018). Furthermore, we aim to understand the regulatory network governing their expression profiles in response to various external stimuli Mtb encounters during host infection, such as nutrient depletion, hypoxia, and antibiotic exposure. We believe that the information in this mini-review will aid in assessing whether specific Mtb CYPs and redox partners are suitable candidates for the development of novel anti-TB drugs.

### The CYP family and cognate redox partners in Mtb

The 4.4 Mb genome of Mtb H37Rv contains twenty  *CYP* genes (Cole et al. 1998). This is a relatively high number for prokaryotes, as most sequenced bacterial genomes generally contain a small number of *CYP* genes. The high abundance of *CYP* genes is a general characteristic of mycobacteria exemplified by *Mycobacterium rhodesiae NBB3* (70), *Mycobacterium avium 104* (48), *Mycobacterium marinum* (47), *Mycobacterium smegmatis MC2* (42), and *Mycobacterium abscessus 47J26s/103* (25) as compiled by Parvez et al. (2016). In contrast, *Mycobacterium leprae* has lost all but one functional CYP protein, encoded by *ML2088c* (37% identity to Mtb CYP140A1). However, this is consistent with the overall reduction in genome size in *M. leprae*. Deciphering the physiological function of CYPs is usually performed through a combination of homology modeling/mapping, generation of single gene knockouts and/or biochemical characterization of the protein. Yet, despite the high abundance of CYP genes in mycobacteria, phylogenetic analysis has shown that Mtb CYPs exhibit relatively low evolutionary relationships with other species, with many bacterial species (like *Escherichia coli*) lacking CYPs altogether. CYP121A1, CYP128A1, CYP135, and CYP141A1 are predominantly found in mycobacteria belonging to the MTBC (Ouellet et al. 2010a). In addition, the overall homology between the 20 Mtb CYPs is rather low, with a maximum homology of 40% between CYP135A1 and CYP135B1 in Mtb.

The catalytic activity of CYPs is dependent on their redox partners (McLean et al. 2005). CYPs are classified based on the type of redox systems they pair with. All Mtb CYPs belong to class I, meaning their catalytic activity is dependent on a NAD(P)H-ferredoxin-reductase (FNR) and a ferredoxin (Fd). Mtb encodes five ferredoxins genes, Fdx (*Rv0763c*), FdxA (*Rv2007c*), FdxC (*Rv1177*), FdxD (*Rv3503c*), and Rv1786; two FNRs namely FdrA (*Rv0688*) and FprA (*Rv3106*); and two Fd-FNR fusions, FdxB (*Rv3554*) and FprB (*Rv0886*). Fdx, FdxD, and FdxE share a common Cys-*X*_2_-*X*-*X*_2_-Cys-*X*_*n*_-Cys-Pro iron-sulfur cluster-binding motif, where *X* is any amino acid and *X*_*n*_ indicates a number of flexible amino acids. This motif is consistent with a [3Fe-4S] cluster which is conserved on ferredoxins from other bacteria (Trower et al. 1993; Duff et al. 1996; Green et al. 2003; Sevrioukova 2005; Lu et al. 2017; Child et al. 2018). On the other hand, FdxA and FdxC resemble FdxA from *M. smegmatis* (*MsFd*), a 7Fe-ferredoxin that contains both a [3Fe-4S] and a [4Fe-4S] iron-cluster combined in a single protein (Ricagno et al. 2007; Ortega Ugalde et al. 2018a). The overall sequence identity between the [3Fe-4S] cluster-containing Mtb ferredoxins Fdx, FdxD, and FdxE is approximately 30%, whereas FprA displays approximately 40% sequence identity to the mammalian adrenoxin reductase (AdR) (Ricagno et al. 2007; Fischer et al. 2002; Ortega Ugalde et al. 2018a).

### Mtb CYPs and cognate redox partners essentiality

Important information on the essentiality of certain CYPs for bacterial adaptation, growth, survival, and virulence can be obtained from transposon site hybridization (TraSH) studies. To date, several of these studies have been performed and a summary of the results for Mtb CYPs and their cognate redox partners is presented in Table 1.

**Table 1:** Essentiality and conditional essentiality of Mtb CYPs and cognate redox partners

Protein	Gene	Essential (in vitro)					Conditionally essential for survival					
		Himar-1 transposon H37Rv strain^a^	Himar-1 transposon H37Rv strain^b^	Himar-1 transposon H37Rv strain^c^	Himar-1 transposon CDC1551 strain^d^	Himar-1 transposon H37Rv strain^e^	Cholesterol^f^	Macrophages^g^	Mice spleen^h^	Mice lungs^i^	Guinea pigs^j^	Primates^k^
CYPs												
CYP121A1	*Rv2276*	N	N	N	N	ND	N	N	N	N	N	N
CYP123A1	*Rv0766c*	N	N	N	N	N	N	N	N	N	N	N
CYP124A1	*Rv2266*	N	N	N	N	N	N	N	N	N	N	N
CYP125A1	*Rv3545c*	N	N	N	N	N	Y	N*	Y	N	N	N
CYP126A1	*Rv0778*	N	N	N	N	ND	N	N	N	N	N	N
CYP128A1	*Rv2268c*	N	N	N	N	Y	N	N	N	N	N	N
CYP130A1	*Rv1256c*	N	D	N	N	ND	N	N	N	N	N	N
CYP132A1	*Rv1394c*	N	N	N	N	N	N	N	N	N	N	N
CYP135A1	*Rv0327c*	N	N	N	N	ND	N	N	N	N	N	N
CYP135B1	*Rv0568*	N	N	N	N	N	N	N	N	N	N	N
CYP136A1	*Rv3059*	GA	N	N	N	N	N	N	N	N	N	N
CYP137A1	*Rv3685c*	N	N	N	N	N	N	N	N	N	N	N
CYP138A1	*Rv0136*	N	N	N	N	N	N	N	N	N	N	N
CYP139A1	*Rv1666c*	N	N	N	N	N	N	N	N	N	N	N
CYP140A1	*Rv1880c*	N	N	N	N	N	N	N	N	N	N	N
CYP141A1	*Rv3121*	N	N	N	N	ND	N	N	N	N	N	N
CYP142A1	*Rv3518c*	N	N	N	N	N	N	N	N	N	N	N
CYP143A1	*Rv1785c*	N	N	N	N	N	N	N	N	N	N	N
CYP144A1	*Rv1777*	N	D	N	N	N	N	N	N	N	N	N
CYP51B1	*Rv0764c*	N	N	N	N	N	N	N	N	N	N	N
Redox partners												
Fdx	*Rv0763c*	Uncertain	Short	N	N	ND	N	N	N	N	N	N
FdxA	*Rv2007c*	N	N	N	N	ND	N	N	N	N	N	N
FdxB	*Rv3554*	N	D	N	N	N	N	N	N	N	N	N
FdxC	*Rv1177*	Y	Short	N	N	Y	N	N	N	N	N	N
FdxD	*Rv3503c*	N	N	N	N	N	N	N	N	N	N	N
FdxE	*Rv1786*	Uncertain	N	N	N	N	N	N	N	N	N	N
FdrA	*Rv0688*	N	N	N	N	N	N	N	N	N	N	N
FprA	*Rv3106*	N	N	N	N	N	N	N	N	N	N	N
FprB	*Rv0886*	N	N	N	N	N	N	N	N	N	N	N

*N* non-essential; *Y* essential; *ND* no data available; *D* contains both required and non-required regions; *Short* too short to call, no insertions; *GA* growth advantage

*Gene part of a putative operon required for survival in macrophages

^a^DeJesus et al. 2017

^b^Zhang et al. 2012

^c^Griffin et al. 2011

^d^Lamichhane et al. 2003

^e^Sassetti et al. 2003

^f^Griffin et al. 2011

^g^Rengarajan et al. 2005

^h^Sassetti and Rubin 2003

^i^Lamichhane et al. 2005

^j^Jain et al. 2007

^k^Dutta et al. 2010

The results of these studies confirm the non-essentiality of individual Mtb CYP enzymes for in vitro growth under standard laboratory conditions. This is not surprising since a high number of genes found to be essential for in vitro growth were conserved in the degenerate genome of the leprosy bacillus, *M. leprae* (Sassetti et al. 2003). Even though an initial study indicated that *cyp128a1* was among the essential genes unique to mycobacteria, this could not be confirmed in later TraSH studies (Sassetti et al. 2003; Lamichhane et al. 2003; Griffin et al. 2011; Zhang et al. 2012; DeJesus et al. 2017). These contradictory results could be due to the limited gene coverage of some of the transposon libraries. Interestingly, disruption of the orphan cyp*136a1* provided a growth advantage in vitro (DeJesus et al. 2017). Even though *cyp121a1* was found to be non-essential in these genome-wide transposon studies, *cyp121a1* could only be knocked out in Mtb H37Rv after creating a merodiploid strain. This indicates that this gene is required for growth under in vitro conditions (McLean et al. 2008).

Transposon insertion studies were also conducted on bacterial cultures grown in conditions that the bacterium encounters during infection in the human host. These studies aimed to identify the mechanisms used by the bacterium to resist these insults. Griffin et al. identified *cyp125a1* as the sole monooxygenase essential for Mtb H37Rv growth in cholesterol, an essential carbon source during Mtb infection. Furthermore, *cyp125a1* was found to be essential during mouse infection (Sassetti and Rubin 2003). Although *cyp125a1* was not found to be required for macrophage infection, it is part of the *fadE28* locus, which is a critical operon involved in lipid transport, lipid degradation and assimilation of exogenous lipids from host cell membranes (Cole et al. 1998; McKinney et al. 2000; Rengarajan et al. 2005).

Both FdxA and FdxC (the two 7Fe-ferredoxins) were found to be essential for Mtb in vitro (Sassetti et al. 2003; DeJesus et al. 2017). For FdxA, essentiality was predicted using a pathway enrichment method (Xu et al. 2014). Several transposon mutagenesis studies indicate that individual redox partners may not be essential under certain growth conditions, such as cholesterol rich conditions or during infection in vivo (Lamichhane et al. 2003; Sassetti et al. 2003; Sassetti and Rubin 2003; Lamichhane et al. 2005; Rengarajan et al. 2005; Jain et al. 2007; Dutta et al. 2010; Griffin et al. 2011; Zhang et al. 2012; DeJesus et al. 2017). These results could be explained by redundancy of redox partners in CYP-mediated reactions previously shown not only for Mtb (Ortega Ugalde et al. 2018a) but also for other bacteria, such as *M. marinum*, *Sorangium cellulosum So ce56* and *Streptomyces peucetius DoxA* (Ewen et al. 2009; Rimal et al. 2015; Child et al. 2018).

### Mtb CYPs and cognate redox partners function

Only a handful of the 20 Mtb CYPs and cognate redox partners have been structurally and biochemically characterized. In this section, we summarize the current knowledge of the (partially) characterized Mtb CYP enzymes and associated redox partners. For a more elaborate discussion, see also (McLean et al. 2006a, 2007, 2010; McLean and Munro 2008; Ouellet et al. 2010a; Ortiz de Montellano 2018).

#### CYP51B1: the first prokaryotic sterol demethylase

**Fig. 1:**
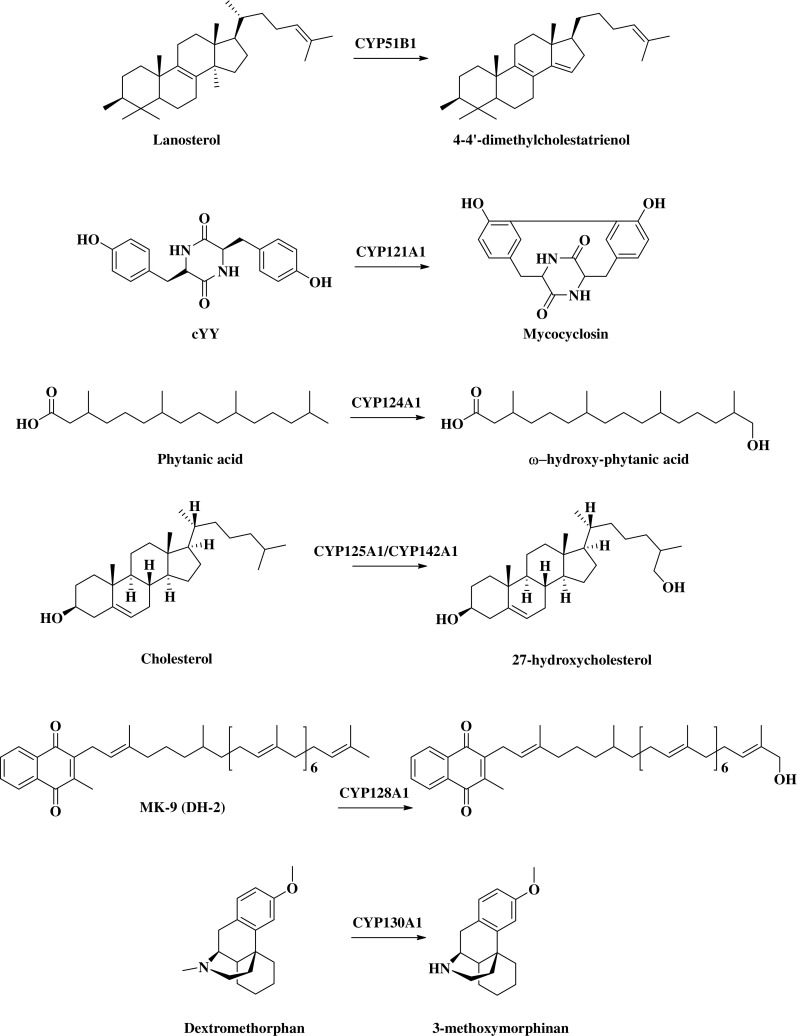
Substrates and reactions for Mtb CYP enzymes. CYP51B1 sterol demethylase catalyzes oxidative removal of 14α-methyl group from lanosterol, dihydrolanosterol, and obtusitol (Bellamine et al. 1999). CYP121A1 catalyzes the oxidative coupling of the two tyrosyl atoms (Belin et al. 2009). CYP124A1 catalyzes the ω-hydroxylation of phytanic acid and other methyl branched-chains fatty acids (Johnston et al. 2009). CYP125A1 and CYP142A1 catalyze the 27-hydroxylation of cholesterol (McLean et al. 2009; Capyk et al. 2009; Driscoll et al. 2010; Ouellet et al. 2010a). CYP130A1 catalyzes N-demethylation of dextromethorphan (Ortega Ugalde et al. 2018). CYP128A1 catalyzes the oxidative hydroxylation of menaquinone MK-9 (DH-2) (Holsclaw et al. 2008)

*Cyp51b1* was the first prokaryotic sterol demethylase gene identified in a bacterial genome. Spectroscopic analysis of the recombinant CYP51B1 showed that the P450 form rapidly converted to the P420 species. The stability of the P450 form was increased when it was bound to its cognate substrate analog estriol (McLean et al. 2006b; Dunford et al. 2007). CYP51B1 was the first Mtb CYP enzyme for which crystal structures were unraveled in the ligand-free and ligand-bound states. The enzyme was co-crystalized with azole compounds. Azoles were shown to bind with high affinity to CYP51B1, with a *K*_*D*_ equal to 0.18, 0.2, 0.3, and 2.1 μM for clotrimazole, miconazole, econazole, and voriconazole, respectively (Aoyama et al. 1998; Podust et al. 2001, 2004). Mtb CYP51B1 displayed sterol demethylase activity with lanosterol and dihydrolanosterol as substrates. In addition, the plant sterol obtusifoliol was converted to the respective 8,14-diene products when reconstituted with cognate and surrogate bacterial redox partners (Fig. 1) (Bellamine et al. 1999; Zanno et al. 2005). However, the relevance of this activity to mycobacterial physiology remains unclear, since the complete sterol biosynthetic pathway is lacking in Mtb (McLean and Munro 2008). The unknown physiological role combined with the non-essentiality may limit the suitability of CYP51B1 as a drug target in Mtb.

#### CYP121: bacterial secondary metabolism

CYP121A1 catalyzes the formation of a carbon-carbon bond between the two tyrosyl carbon atoms of a cyclodipeptide cyclo(L-Tyr-L-Tyr) (cYY) yielding mycocyclosin (Fig. 1) (Belin et al. 2009). The physiological role of mycocyclosin for Mtb remains unknown. However, several cyclic dipeptides have been shown to have an impact on bacterial physiology. For instance, the cyclo(Phe-Pro) inhibited the production of virulence factors in *Vibrio cholerae* (Park et al. 2006; McLean et al. 2008, 2010). CYP121A1 is considered a highly selective CYP, since it does not efficiently metabolize any cYY analogs (Fonvielle et al. 2013). Spectroscopic characterization of recombinant CYP121A1 showed the same P450 collapse of the CO-ferrous state as previously described for CYP51B1. However, this event was reversible at lower pH, suggesting that the state was dependent on the protonation of the cysteine thiolate (Cys345) to thiol (Dunford et al. 2007). CYP121A1 binds with high affinity to azole drugs (*K*_*D*_ equal to 0.027, 0.073, 0.136, and 8.61 μM for econazole, clotrimazole, miconazole and fluconazole, respectively (McLean et al. 2002a, b; Seward et al. 2006). CYP121A1-fluclonazole crystal structure (PDB 2IJ7) demonstrates that azole coordination of P450 heme can occur via a bridging water molecule, as well as directly to the iron (Seward et al. 2006).

#### CYP124A1, CYP125A1, and CYP142A1: the cholesterol and fatty acid monooxygenase CYPs

CYP124A1 catalyzes ω-hydroxylation reactions with a significant preference for methyl-branched lipids (Fig. 1) (Johnston et al. 2009). Several studies have shown that CYP124A1, CYP142A1, and CYP125A1 are involved in the successive oxidations of the aliphatic side chain of cholesterol and cholest-4-en-3-one at carbon position 27 to the alcohol, aldehyde, and the carboxylic acid (Fig. 1) (Johnston et al. 2010). CYP126A1 may have a related function/activity, as it shows a 37%, 36%, and 32% amino acid sequence homology with CYP124A1, CYP125A1, and CYP142A1, respectively (Ouellet et al. 2010a). CYP126A1 showed a strong preference for the binding of aromatics and chlorophenol moieties (Hudson et al. 2014). In the presence of surrogate redox partners from *Spinacea oleracea*, CYP124A1, CYP142A1, and CYP125A1 oxidized the cholesterol side chain to carboxylic acid. This reaction is a prerequisite for the entry of cholesterol into the β-oxidation pathway (Johnston et al. 2010). However, this sequential metabolic activity was not seen when several cognate redox partners were used to reconstitute the system (Ortega Ugalde et al. 2018a). CYP125A1 is the most efficient catalyst, followed by CYP142A1 and finally CYP124A1 (Johnston et al. 2010; Ouellet et al. 2010b). Utilization of host cholesterol as a carbon source enables Mtb to persist in macrophages and survive the harsh environment of the granuloma inside the human lung (Ouellet et al. 2011; Lee et al. 2013; Wilburn et al. 2018). Knockout of the intracellular growth (*igr*) locus (from *Rv3545c* to *Rv3540c*, which includes *cyp125a1*) led to accumulation of cholest-4-en-3-one, which not only prevented the growth of Mtb in cholesterol-rich medium, but also when grown with acetate, glucose and glycerol as sole carbon source (Chang et al. 2007, 2009; Frank et al. 2016). Hence, targeting CYPs involved in cholesterol catabolism could be a contribution to the antitubercular therapy (VanderVen et al. 2015).

#### CYP128A1: a possible role in virulence

Sassetti et al. initially identified *cyp128a1* as essential for Mtb growth in vitro*.* Subsequent transposon mutagenesis studies did not validate this essentiality (Table 1). Several attempts to heterologously express CYP128A1 have been unsuccessful, preventing its biochemical and structural characterization. The operon containing *cyp128a1* is comprised of three genes involved in the biosynthesis of a sulfated metabolite, S881, also known as sulfomenaquinone. It was hypothesized that CYP128A1 catalyzes the required hydroxylation of dihydromenaquinone (MK-9 DH2), which is further sulfonated by a sulfotransferase (Sft3 encoded by *Rv2267c*) yielding S881 (Fig. 1) (Cole et al. 1998; Holsclaw et al. 2008). S881 is a sulfolipid (SL-1) located in the outer membrane cell wall, where it acts as negative regulator of virulence (Mougous et al. 2006; ten Bokum et al. 2008; Jackson 2014). Hence, assuming that CYP128A1 is indeed a quinol hydroxylase, it is an unlikely therapeutic target for Mtb.

#### CYP130A1: an orphan enzyme

*Cyp130a1*, as well as *cyp141a1*, are not required for Mtb growth in vitro. Furthermore, these genes are absent in the closely related species *Mycobacterium bovis*, suggesting that, if they play a role in virulence, this must be specific for Mtb. *Cyp130a1* is located alongside the same functional operon as *Rv1258c*, a gene encoding a putative tetracycline/aminoglycoside resistance (TAP)2-like efflux pump, which is upregulated treatment with tetracycline, streptomycin, erythromycin, and rifampicin (unlike *cyp130a1*, as discussed in section 5) and has been associated with efflux-mediated resistance in MDR-TB (Siddiqi et al. 2004; Morris et al. 2005; Jiang et al. 2008; Burian et al. 2012; Boot et al. 2018). Yet, no direct functional association has been reported between *cyp130a1* and *Rv1258c*. Recently, it was shown that CYP130A1 can catalyze the N-demethylation of dextromethorphan yielding 3-methoxymorphinan (Fig. 1) (Ortega Ugalde et al. 2018b). Although dextromethorphan cannot be considered a physiological substrate for this enzyme, it does provide a starting point for (1) the identification of bacterial reactions CYP130A1 may catalyze and (2) the identification of inhibitors. Nevertheless, because of the lack of comprehensive data on the impact of CYP130A1 on Mtb virulence, its potential as a drug target remains to be determined.

#### CYP139A1: a possible host-response modulator and/or detoxifying enzyme

CYP139A1 (*Rv1666c*) is conserved across different mycobacterium species, such as *M. marinum* (*MMAR2475*), *M. bovis* (*Mb1694c*), and *M. leprae* (*ML1238c*, pseudogene), as part of a cluster consisting of polyketide synthase genes (*pks11*, *9*, and *17*) and a macrolide transport ATP-binding protein ABC transporter gene. Polyketides are lipid-like molecules that are smaller than known protein toxins but have potent biological activities, such as, antibiotic (erythromycin), immunosuppressant (rapamycin), and antifungal (amphotericin B). In Mtb, the majority of the PKS-encoding genes have been linked to specific biosynthetic pathways required for the production of unique lipids or glycolipid conjugates that are critical for virulence and/or components of the organized outer membrane of the mycobacterial cell envelope. As an example, Mtb was shown to be capable of altering essential host functions by the release of polyketides that can induce a G0/G1 cell cycle arrest in the macrophages. As a result, mycobacteria are capable of challenging innate killing mechanisms and evade the adaptive immune system as non-cycling cells are less likely to be killed by cytotoxic T cells (Nishioka and Welsh 1994; Cumming et al. 2017). *Pks9* is an immunogenic protein (Kumar et al. 2011), whereas *pks17* (together with *pks8*, *Rv1662)* is involved in the production of methyl-branched fatty acids which are minor components of sulfolipids and acyltrehaloses (Dubey et al. 2003). *Pks11* could be involved in the assembly of alkylpyrones from fatty acyl-CoA and malonyl-CoA (Saxena et al. 2003; Gokulan et al. 2013), but the production of long-chain α-pyrone metabolites in Mtb has not been shown. Hence, CYP139A1 could play a conserved role in the modification and/or transport of these *Psk*-derived molecules and it could be associated with the host-pathogen interactions. On the other hand, the observation that CYP139A1 is induced after exposure to antibiotics (see below) is intriguing and may suggest a role in detoxification.

#### CYP143A1: component of the ESX-5 secretion locus

The gene encoding the orphan enzyme CYP143A1 lies adjacent to *Rv1786* encoding FdxE. This proximity suggests a CYP/redox partner association. Indeed, a significant binding affinity between FdxE and CYP143A1 was demonstrated with a *K*_*D*_ value equal to 10^−7^ M (Lu et al. 2017). In Mtb and several closely related species, *cyp143a1* is part of the *esx-5* locus. Most genes of this *esx-5* locus are coding for substrates or components of the ESX-5 protein secretion machinery. This ESX-5 secretion system completes a multitude of functions, linked to the many different Pro-Glu (PE) and Pro-Pro-Glu (PPE) protein substrates of the system. Some of these proteins remain in the cell envelope, where they are important for modifying the cell permeability and uptake of hydrophobic carbon sources (Abdallah et al. 2009; Ates et al. 2015). In addition, Mtb ESX-5 system mediates the reduction of pro-inflammatory cytokine secretion by macrophages and induces a caspase-independent cell death in macrophages after phagosomal escape of the bacteria enabling the bacteria to infect neighboring cells (Abdallah et al. 2008, 2011). Also, ESX-5 components are upregulated during phosphate-limiting conditions (Elliot and Tischler 2016). Even though the specific role of *cyp143a1* remains unclear, its association with the *esx-5* locus may reflect a pivotal contribution to components of the transport channel. Furthermore, ESX-5 transposon insertion mutants in the gene encoding CYP143A1 Mtb were attenuated both in macrophages and in the immunodeficient mouse infection model (Bottai et al. 2012; Sayes et al. 2012). The same holds true for MMAR_2666 (*cyp143a1* homolog in *M. marinum*) during infection in RAW, THP-1, or CLC cell lines (Weerdenburg et al. 2015). These results confirm that CYP143A1 might have a conserved pathogenic role and therefore highlights its potential use as a drug target.

#### Cognate redox partners for Mtb CYPs

The relatively low number of redox partners compared to the number of *CYP* genes in Mtb implies that these redox proteins are promiscuous in mediating electron transfer. However, some CYPs may have evolved with protein binding sites that are specific for (a) certain redox partner(s). Recently, it was shown that the activity of a small selection of Mtb CYPs was supported by several cognate redox partners in vitro (Ortega Ugalde et al. 2018a). Both Fdx and FdxD supported CYP121A1 catalytic activity with comparable efficiencies whereas FdxD and FdxE supported in vitro reconstitution of cholesterol hydroxylation by Mtb CYP124A1, CYP125A1, and CYP142A1, although with lower efficiency for FdxE. Catalytic efficiencies were equal for the two different FNRs (FdrA and FprA), indicating a flexible CYP/FNR/Fd catalytic system in vivo. Previous studies have shown the ability of the FprA-Fdx redox system to reduce CYP51B1 in vitro (McLean et al. 2006a). Moreover, the FdrA-Fdx complex was capable of supporting CYP51B1 activity through the same electron transfer system (Zanno et al. 2005). FdxA was the only ferredoxin unable to transfer electrons to any of the studied CYPs (Ortega Ugalde et al. 2018a). In addition, FdxC, which has not been successfully expressed yet, is required for optimal growth of Mtb in vitro suggesting a key role in shuttling electrons (Sassetti et al. 2003; DeJesus et al. 2017).

Characterization of the electron transfer capabilities of FdxC, FdxB, and FprB, to (all) Mtb CYPs could contribute to understanding the complex biochemistry of the CYP/FNR/Fd network.

### Mtb CYPs and cognate redox partners expression levels

Mtb encounters a number of physiological stresses that trigger coordinated stress responses orchestrated by protein expression regulation. A summary of transcriptome analyses under different insults focusing on Mtb CYPs and their cognate redox partners is presented in Table 2. Induced expression as part of the Mtb adaptive response could indicate an important role in survival.

**Table 2:** Expression levels of Mtb CYPs and cognate redox partners

Protein	Gene	Stress									
		Stationary phase^a^	Starvation^b^	pH stress^c^	NO treatment^d,e^	CO treatment^f^	Hypoxia^g,h,i^	High temperature^j^	Phagocytosis^k^	DNA damage^l^	Salicylate treatment^m^
CYPs											
CYP121A1	*Rv2276*	↓	↑	–	↓	–	–	–	–	–	–
CYP123A1	*Rv0766c*	–	↑	–	–	–	–	↑	–	–	–
CYP124A1	*Rv2266*	–	–	–	–	–	–	–	–	–	–
CYP125A1	*Rv3545c*	–	↑	–	–	–	–	–	–	–	–
CYP126A1	*Rv0778*	–	↑	–	–	–	–	–	–	–	–
CYP128A1	*Rv2268c*	↓	↑	–	–	–	–	–	–	–	–
CYP130A1	*Rv1256c*	↓	–	–	–	–	–	–	–	–	–
CYP132A1	*Rv1394c*	–	↑	–	–	–	–	–	–	–	–
CYP135A1	*Rv0327c*	–	↑	–	–	–	–	–	–	–	–
CYP135B1	*Rv0568*	–	↑	–	–	–	–	–	–	–	–
CYP136A1	*Rv3059*	–	↑	–	–	–	↑	–	–	–	–
CYP137A1	*Rv3685c*	↓	↑	–	–	–	–	–	–	–	–
CYP138A1	*Rv0136*	↓	↑	–	–	–	–	↑	–	–	–
CYP139A1	*Rv1666c*	–	–	–	–	–	–	–	–	–	–
CYP140A1	*Rv1880c*	↓	↑	–	–	–	–	–	–	–	–
CYP141A1	*Rv3121*	–	↓	–	–	–	–	–	–	–	–
CYP142A1	*Rv3518c*	–	↑	–	–	–	–	–	–	–	–
CYP143A1	*Rv1785c*	↓	–	–	–	–	–	–	–	–	–
CYP144A1	*Rv1777*	↓	–	–	–	–	–	–	–	–	–
CYP51B1	*Rv0764c*	–	↑	–	–	–	–	–	–	–	–
Redox partners											
Fdx	*Rv0763c*	–	–	–	–	–	–	–	–	–	–
FdxA	*Rv2007c*	–	↓	↑	↑	↑	↑	↑	↑	–	–
FdxB	*Rv3554*	–	–	–	–	–	–	–	–	–	–
FdxC	*Rv1177*	–	↓	–	–	–	–	–	–	–	–
FdxD	*Rv3503c*	–	↑	–	–	–	–	–	–	–	–
FdxE	*Rv1786*	–	–	–	–	–	–	–	–	–	–
FdrA	*Rv0688*	–	↑	–	–	–	–	–	–	–	–
FprA	*Rv3106*	–	–	–	↓	–	–	–	–	–	–
FprB	*Rv0886*	↑	–	–	–	–	↑	–	–	–	–

^a^Hampshire et al. 2004

^b^Betts et al. 2002

^c^Fisher et al. 2002

^d^Voskuil et al. 2003

^e^Namouchi et al. 2016

^f^Shiloh et al. 2008

^g^Sherman et al. 2001

^h^Bacon et al. 2004

^i^Voskuil et al. 2004

^j^Stewart et al. 2002

^k^Schnappinger et al. 2003

^l^Namouchi et al. 2016

^m^Denkin et al. 2005

#### Stress response

**Fig. 2:**
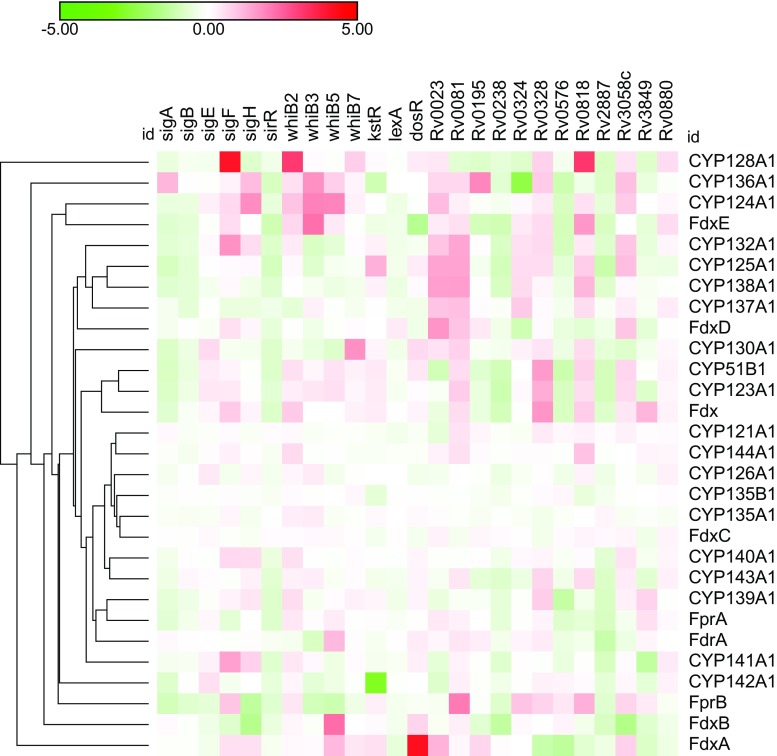
Two-dimensional hierarchical clustering of Mtb CYPs and cognate redox partners expression profiles after transcription factor overexpression. The individual genes are represented on the *x*-axis and the transcription factors are indicated on the *y* axis. Red indicates upregulation whereas green indicates downregulation and white indicates no change relative to time zero control. Data retrieved from Rustad et al. 2014

During latent infection Mtb is thought to survive in granulomas by entering a non-replicating state (Lenaerts et al. 2015). This condition is the bacterial response to a number of different stimuli, such as lack of nutrients, oxygen depletion, and high concentrations of NO (Betts et al. 2002; Voskuil et al. 2004; Bacon et al. 2004). Transcriptomic studies conducted under conditions that stimulate nutrient depletion have revealed that from the Mtb CYP complement (CYPome), only the expression of *cyp128a1* was upregulated, whereas the remaining CYPs showed a minimal alteration in their expression levels (Betts et al. 2002). *cyp128a1* induction was also observed upon overproduction of the transcriptional regulators (TRs) WhiB2 and SigF, which are known to be induced as a result of nutrient depletion and thought to be involved in promoting bacterial survival and proliferation during infection in lung granulomas (Fig. 2) (Sherman et al. 2001; Bacon et al. 2004; Voskuil et al. 2004; Rustad et al. 2014). Only *cyp136a1* expression was upregulated in response to hypoxic conditions (Sherman et al. 2001; Bacon et al. 2004; Voskuil et al. 2004). Likewise, *cyp136a1* upregulation was also seen by overproduction of WhiB3, a redox-sensitive TR responsive to NO and O_2_ as well as upon overproduction of Rv0195, a LuxR transcription factor involved in bacterial virulence in human macrophage-like cells and murine tissues (Fig. 2) (Fang et al. 2013; Rustad et al. 2014). TRs Rv0238 and Rv3058c belong to the TetR family and both regulate efflux pumps and transporters, which are involved in antibiotic resistance and confer tolerance against toxic compounds (Cagliero et al. 2005; Guazzaroni et al. 2005; Lin et al. 2005; Akiba et al. 2006). Interestingly, overproduction of these two TRs yielded the transcriptional induction of several Mtb CYPs and associated redox partners, such as *cyp128a1*, *cyp136a1*, *cyp143a1*, *cyp136a1*, *Fdx*, and *FdxA*, suggesting an involvement in antibiotic resistance pathways (Fig. 2) (Rustad et al. 2014). In contrast, the expression of virtually all Mtb CYPs and redox partners genes was downregulated in the stationary phase following progressive nutrient depletion, which could be explained by the lower transcriptional activity mycobacteria undergo under nutrient starvation (Betts et al. 2002; Hampshire et al. 2004). The number of transcripts of FdxA was increased in all conditions known to induce the dormancy regulon (DosR) regulon, especially by reduced O_2_ tension, which induces a switch to anaerobic metabolism (Sherman et al. 2001). In addition, FdxA is upregulated upon activation of the DosR regulon, which is comprises a set of genes that is essential for the pathogen’s ability to persist during lengthy hypoxia and to rapidly recover from non-respiratory dormancy (Fig. 2) (Rustad et al. 2014). Only FdxA showed an increased expression after pH stress, phagocytosis, and carbon monoxide (CO) treatment (Table 2). There was also a significant change in its expression level during mycobacterial growth in the immune-competent (BALB/c) and the severe combined immune-deficient (SCID) mice (Fischer et al. 2002; Voskuil et al., 2003; Talaat et al. 2004; Shilou et al. 2008; Namouchi et al. 2016).

Overproduction of KstR, a highly conserved TR that regulates a large set of gene-encoding proteins involved in cholesterol catabolism, resulted in a high induction of *cyp125a1*. In contrast, the other CYPs related to cholesterol metabolism were downregulated (*cyp142a1*) or unchanged in expression (*cyp124a1* and *cyp126a1*) (Fig. 2) (Rustad et al. 2014). These results are in line with the reported induction levels of *cyp142a1* in cholesterol-rich growth conditions (Johnston et al. 2010). These results suggest that even though CYP142A1 is able to catalyze cholesterol oxidation, CYP125A1 is the main monooxygenase involved in this pathway.

Bacteria respond to DNA damage by mounting a coordinated stress response; the so-called SOS response. This response is governed by the DNA repair genes *recA* and its cognate repressor *lexA*. The Mtb CYPs and their cognate electron-transfer partners are not part of the SOS box response. Therefore, it is not surprising that their expression levels remained unchanged upon DNA damage or in response to overproduction of LexA (Table 2 and Fig. 2) (Little 1991; Davis et al. 2002; Rustad et al. 2014; Namouchi et al. 2016).

A number of TRs mediate the regulation of genes involved in resistance to a diverse array of antibiotics that target different pathways in Mtb. For example, SigB is specifically involved in tolerance to ethambutol and isoniazid, whereas *sigE* null mutant strains are more sensitive to vancomycin, rifampin, streptomycin, gentamicin, isoniazid, and ethambutol (Pisu et al. 2017). The TRs *Rv0324* and *Rv0880* comprise the tolerance network to the recently marketed antitubercular drug, bedaquiline (Peterson et al. 2016). On the other hand, WhiB7 shows a more generic spectrum controlling the expression of a plethora of antibiotic resistant genes, such as *tap* (*Rv1258c*) coding for an efflux pump that provides low-level resistance to aminoglycosides and tetracycline or *erm* (*Rv 1988*) homologous to ribosomal methyltransferases and conferring MLS (macrolide, lincosamide, and streptogramin) resistance (De Rossi et al. 2002; Buriankova et al. 2004; Burian et al. 2013). Figure 2 shows that overproduction of SigB and SigE had little effect on expression levels of Mtb CYPs and redox partners. In contrast, *cyp130a1* was slightly upregulated. The same holds true for overproduction of the TRs *Rv0324* and *Rv0880* (both involved in bedaquiline tolerance) which are both result in upregulation of c*yp128a1*, *cyp137a1*, *fdxE*, and *fprB*. Moreover, overproduction of WhiB7 led to increased transcripts of *cyp128a1*, *cyp130a1*, and *fdxA*, suggesting that they are involved in the dealing with the stress consequent to bedaquiline exposure (Fig. 2) (Rustad et al. 2014). Interestingly, expression of some of these TRs, such as whiB7, is upregulated by antibiotic treatment, as well as by certain (physiological) stresses including iron starvation and heat shock (Geiman et al. 2006).

Figure 2 depicts the TRs that are involved in the expression of Mtb CYP and redox partners (Rustad et al. 2014). Little is known about several TRs depicted in Fig. 2, such as Rv0081 and Rv0818; however, it is clear that they play a role in CYP biology and are possibly involved in drug tolerance.

#### Antibiotic exposure response

**Fig. 3:**
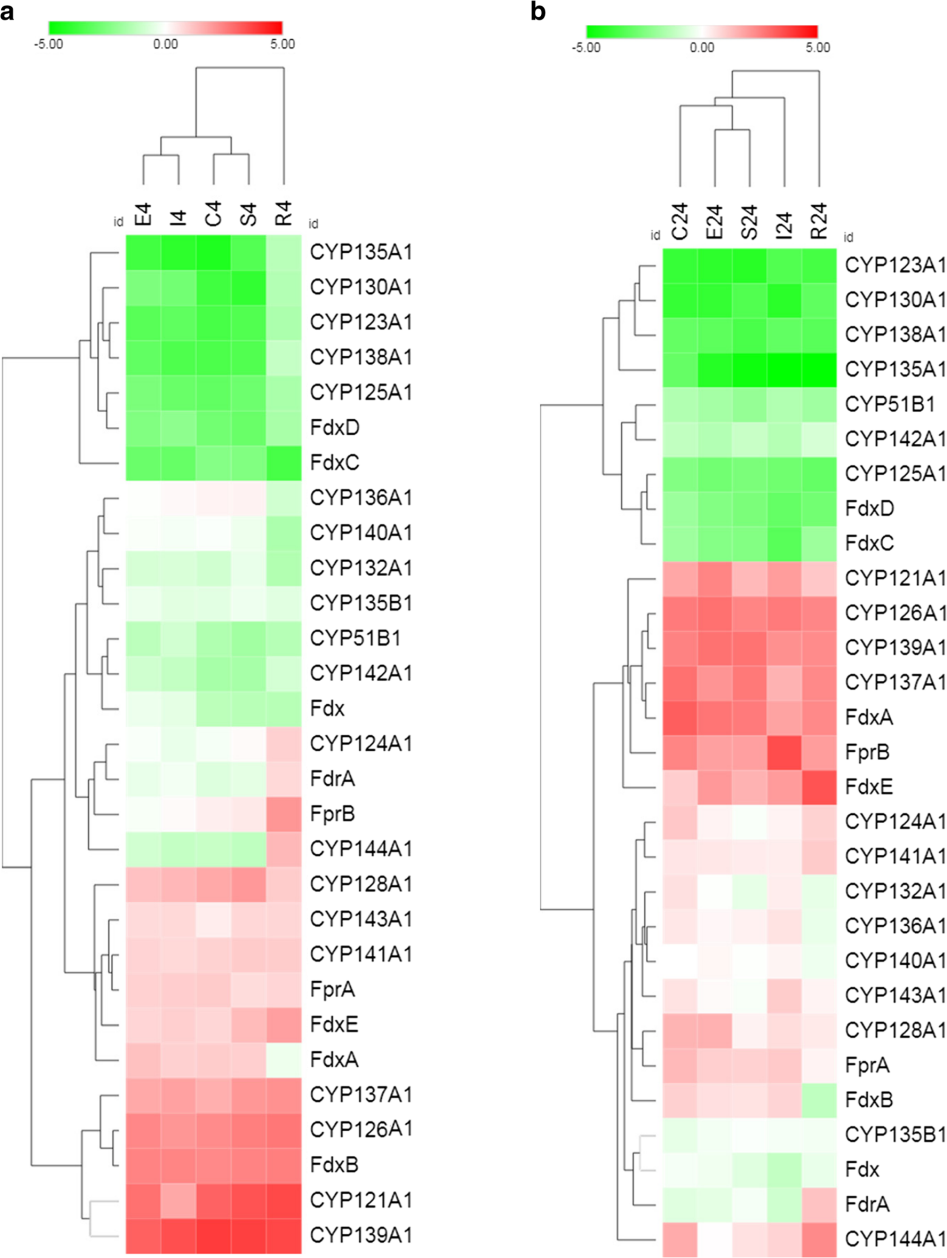
Two-dimensional hierarchical clustering of Mtb CYPs and cognate redox partners expression profiles in response to first- and second-line antibiotic exposure. **a** After 4-h exposure and **b** after 24-h exposure. The individual genes are represented on the *x*-axis and the antibiotics are indicated on the *y-*axis. Red indicates upregulation whereas green indicates downregulation and white indicates no change relative to time zero control. C = ciprofloxacin, E = ethambutol, I = isoniazid, R = rifampicin, S = streptomycin. Data retrieved from Boot et al. 2018

Analysis of the transcriptome response of Mtb to antibiotics is a powerful approach to unveil the pathways contributing to drug resistance and may provide new insights relevant to anti-mycobacterial drug discovery. Recently, Boot et al. studied the stress-fingerprint of Mtb upon exposure to subinhibitory concentrations of first- and second-line TB antibiotics with known targets (Boot et al. 2018). In Fig. 3, the transcriptional regulation of Mtb CYPs and redox partners after exposure to these compounds for 4 and 24 h is depicted. It was observed that Mtb mounts a global stress response with similar gene induction/repression to antibiotics, regardless of their cellular target. The antibiotics studied included ciprofloxacin, ethambutol, isoniazid, streptomycin, and rifampicin. Ciprofloxacin inhibits DNA unwinding; ethambutol and isoniazid both target the mycobacterial cell wall; streptomycin inhibits ribosomes; and rifampicin, inhibits RNA polymerase. Furthermore, comparison of the transcriptional response of Mtb at the selected two different time points showed virtually identical overlap, suggesting that Mtb CYPs and associated redox partners play a very specific and robust role upon antibiotic stress (Fig. 3a, b).

Clustering the transcriptional response revealed that *cyp139a1*, *cyp121a1*, *cyp126a1*, and *cyp137a1* together with FdxB, was the most prominently upregulated cluster for all five drugs (Fig. 3). Interestingly, and as previously mentioned, *cyp139a1* is immediately downstream of three polyketide synthase (*pks*) genes and upstream of *Rv1667c*, a gene encoding an ATP-binding cassette (ABC) superfamily transporter. The expression of the three *pks* is downregulated except for *pks17* and *pks9* genes (*Rv1663* and *Rv1664*, respectively) upon exposure to ciprofloxacin and rifampicin. In contrast, Rv1667c is also induced upon exposure to first-line antibiotics (Zhang et al. 2017; Boot et al. 2018). Furthermore, it can also be speculated that increased expression of *cyp139a1* (or one of the other CYPs) contributes to the intrinsic resistance of Mtb to anti-TB agents.

*Cyp143a1* and its tentative associated cognate redox partner, *Rv1786* (FdxE), were upregulated after exposure to these antibiotics (Fig. 3) (Boot et al. 2018)*.* Furthermore, the expression of *Rv1790*, encoding PPE27, was also upregulated whereas other genes in the ESX-5 cluster were downregulated or relatively unaffected (Boot et al. 2018). In addition, FdxA was also induced by exposure to anti-TB compounds, suggesting that this protein is a key player in pathways to resist insults Mtb may encounter in vivo (Fischer et al. 2002; Voskuil et al. 2003; Shilou et al. 2008; Namouchi et al. 2016; Boot et al. 2018). The same holds true for FdxB, after exposure to all four antibiotics (Fig. 3a, b).

The cluster with the strongest downregulation of transcription consisted of *cyp135a1*, *cyp130a1*, *cyp123a1*, and *cyp125a1* together with the electron transfers FdxC and FdxD. A plausible explanation could be that these proteins are constitutively highly expressed (Ricagno et al. 2007). CYP125A1 was shown to be essential for Mtb growth in cholesterol. Its downregulation under antibiotic exposure is consistent with the capability of the bacteria to distinguish different stresses, triggering specific transcriptomic responses (Griffin et al. 2011).

The transcriptional response to bedaquiline was opposite to the results presented in Fig. 3 for the classical anti-TB drugs (Peterson et al. 2016). *Cyp121a1*, *cyp126a1*, *cyp137a1*, and *cyp141a1* were significantly downregulated, whereas *cyp125a1* and *FdxE* were clearly upregulated. This alteration in transcriptome response likely reflects the different mechanism of action of bedaquiline, namely inhibition of the F_1_F_0_-ATP synthase of Mtb, preventing any specific response to be mounted. In addition, it could be the result of bedaquiline-induced decreased protein synthesis due to downregulation of ribosomal subunits (Koul et al. 2014).

Due to the absence of correlation between the transcriptional response of the CYPome after overproduction of several TRs (Fig. 2) and after exposure to anti-TB drugs (Fig. 3), it is not possible to identify TRs which are activated upon antibiotic exposure.

### Targeting Mtb CYPs: state of art

**Fig. 4:**
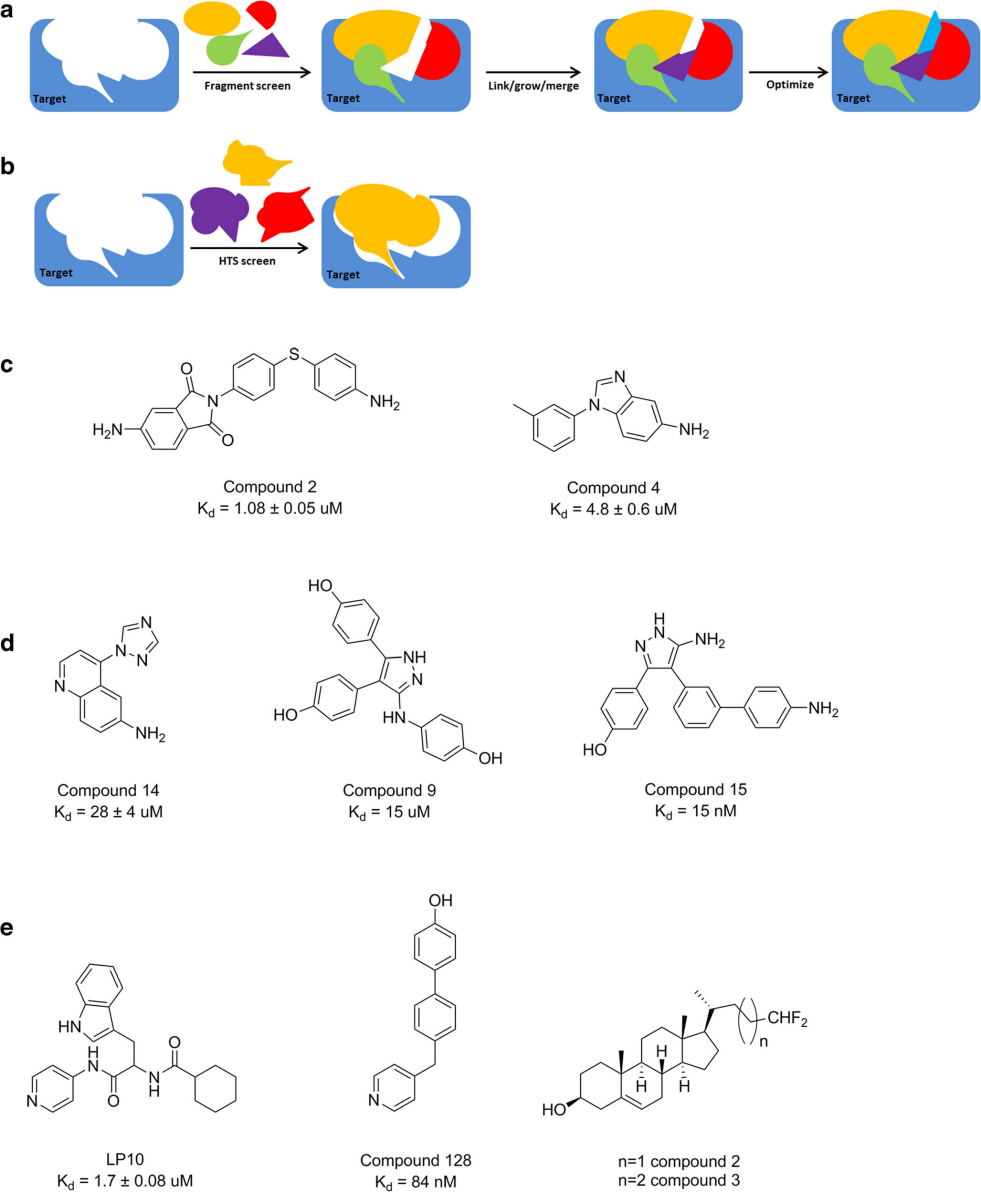
Schematic representation of the strategies for Mtb CYP inhibitors identification. **a** Fragment-based (FB), where fragments are screened against the target of interest, leading to identification of hits, which are linked/grown/merged and finally optimized to generate lead compounds. **b** HTS, where compounds are screened against the target of interest, leading to identification of hit compounds with often lack of optimal interactions with the target. **c** HTS identified high-affinity inhibitor-like binding heterocyclic arylamines for CYP130A1 (Podust et al. 2009). **d** FB identified high-affinity inhibitor-like compounds for CYP121A1 (Hudson et al. 2012, 2013; Kavanagh et al. 2016). **e** HTS identified and specifically synthetized inhibitors for CYP125A1/CYP142A1 (Chen et al. 2009; Ouellet et al. 2011; Brengel et al. 2016; Frank et al. 2016)

Insights into the essentiality of Mtb CYPs and their natural redox partners and the transcriptomic response to external insults are useful to evaluate their potential as therapeutic targets to combat TB. In particular, CYP121A1 and CYP125A1/CYP142A1 are considered promising drug-targets, because several azole-drugs, such as econazole, clotrimazole, and miconazole, bind with high affinities to these Mtb CYPs. Moreover these compounds have been shown to be potent antimycobacterials in vitro, being effective even against persistent and multidrug-resistant strains of Mtb (McLean et al. 2002b; Ahmad et al. 2006a, b; McLean and Munro 2008). In addition, the development of CYP inhibitors relies mostly on high-throughput compound screens (HTS) and fragment-based approaches which provide foundations for a more rational design of selective Mtb CYP inhibitors (Fig. 4a, b). HTS to find potent and highly selective inhibitors of CYP126A1 and CYP130A1 has been performed (Podust et al. 2009; Hudson et al. 2014). In addition, these studies aimed to identify substrates for the orphan CYP126A1 and CYP130A1, which would help to define their physiological roles. This HTS screening on Mtb CYP130A1 identified some inhibitors, belonging to the heterocyclic arylamines which have been shown to cause toxicity issues, deterring their exploitation as antibiotics (Fig. 4c) (Podust et al. 2009; Kim and Guengerich 2005). Furthermore, fragment-based approaches have been applied to CYP121A1 (Hudson et al. 2012; Hudson et al. 2013; Kavanagh et al. 2016). These studies have led to the identification of high affinity type-II binding compounds, with *K*_*D*_ values as low as 15 nM, high Mtb CYP selectivity and low off-target interaction with human CYPs (Fig. 4d) (Kavanagh et al. 2016). However, no significant inhibition of bacterial growth was detected for any of the lead compounds (MIC_90_ ≥ 50 μM) probably due to low membrane permeability and/or increased expression of efflux transporters (Kavanagh et al. 2016).

The CYP inhibitor LP10, initially discovered to block CYP51 from *Trypanosoma cruzi*, showed moderate inhibition of CYP125A1 (Fig. 4e) (Chen et al. 2009; Ouellet et al. 2011). Moreover, screening assays have identified a compound that can inhibit CYP125A1/CYP142A1 (C128), and has a *K*_*D*_ equal to 85 nM (Fig. 4e) (Brengel et al. 2016). Furthermore, two terminally truncated cholesterol analogs with fluorinated side chains were shown to inhibit growth of Mtb (Fig. 4e) (Frank et al. 2016). However, direct inhibition of CYP125A1 and/or CYP142A1 could not be confirmed and the observed growth inhibition was independent of the presence of CYP125A1 (Frank et al. 2016).

## Concluding remarks

New anti-TB drugs are needed to fight resistance and combination therapies provide the most effective treatment of tuberculosis. As such, Mtb CYPs could provide one of the drug targets, as they conduct very specific roles for the pathogen. In addition, their uniqueness to Mtb highlights their attractiveness as therapeutic targets avoiding off-target toxicity. The transcriptional response upon external insults mimicking in vivo situation and drug exposure showed that Mtb CYPs and cognate redox partners might be involved in the adaptation of the pathogen to antibiotics. Further studies will focus on the further biochemical characterization of (orphan) CYPs and redox partners as well as defining the contribution of individual genes by using knockout or knockdown mutants to shed light on their physiological role and their plausible involvement in resistance to antibiotics.
